# Polyvinyl chloride solvent cement poisoning: a case report

**DOI:** 10.1186/s13256-024-04470-x

**Published:** 2024-04-06

**Authors:** S. R. Marambahewa, D. A. C. T Chandrasiri, W. A. I. C. Wijesekara, B. M. Munasinghe

**Affiliations:** 1 District General Hospital, Mannar, Sri Lanka; 2 Ministry of Health, Colombo, Sri Lanka

**Keywords:** S-lon, Solvent cement, Cyclohexanone, Neurotoxicity, Case report

## Abstract

**Background:**

S-lon® (*S*) is a locally produced polyvinyl chloride-based solvent cement. It is a clear, slightly viscous liquid. Other constituents include 1-cyclohexanone, 3-butanone, and 1-acetone. It is used ubiquitously for building construction in Sri Lanka. Although the clinical effects of the compound have not yet been ascertained, the constituents have been implicated in neurotoxicity, respiratory tract, eye and skin irritation, and delayed liver and renal injury.

**Case description:**

A 42-year-old South Asian male presented following self-ingestion of *S*. His vital parameters were stable and initially managed symptomatically. A few hours later, he developed central nervous system depression and stridor requiring elective intubation. Examination of the upper airway revealed inflammation and edema. He was sedated and ventilated, and intravenous dexamethasone was administered. Attempts at removal of the nasogastric tube after extubation on day 3 failed. The patient had to be reintubated and sedated owing to extreme agitation not responding to routine doses of sedatives. The nasogastric tube had been amalgamated after reacting with *S*, forming a solid clump, later found after removal. The posterior pharynx and nasopharynx were packed and later removed before extubation. The patient made a full recovery and was transferred to the ward on day 5.

**Conclusion:**

Ingestion of a sufficient quantity of *S* could result in gut absorption with central nervous system depression, coma, and even death. No antidote is available for toxicity, and management is largely supportive. As witnessed in our patient, chemical laryngitis and upper airway inflammation may lead to upper airway obstruction. Chemical reactions with medical equipment may lead to unforeseen outcomes.

## Introduction

S-lon® (*S*) is a locally produced polyvinyl chloride (PVC)-based solvent cement [1]. It is highly flammable and comes as a clear, slightly viscous liquid. Other constituents include 1-cyclohexanone, 3-butanone, and 1-acetone. It is used ubiquitously for building construction in Sri Lanka. Although the clinical effects of this compound have not yet been ascertained, the constituents have been implicated in neurotoxicity, respiratory tract, eye and skin irritation, and delayed liver and renal injury [1]. Ingestion of a sufficient quantity could result in gut absorption, resulting in central nervous system (CNS) depression, coma, and even death [1]. Deliberate self-ingestion is extremely rare. We report herein a case where a 42-year-old South Asian male presented following self-ingestion of *S* with subsequent CNS depression requiring elective intubation and difficult removal of nasogastric tube (NGT) owing to amalgamation requiring ear, nose, and throat (ENT) input.

## Case description

A 42-year-old South Asian male, a father of two, presented to the emergency treatment unit of a District General Hospital in Sri Lanka following deliberate self-ingestion of *S* following an attempted suicide. No other toxins were found in the vicinity. His past medical, surgical, and allergy history had been insignificant. Admission was within 90 min following the ingestion. On admission, his airway was patent with equal bilateral air entry without added sounds, respiratory rate was 16 breaths per minute with a shallow breathing pattern, and capillary O_2_ saturation was 92% in room air. The pulse rate was 110 bpm; capillary refill was less than 2 s with a blood pressure of 140/85 mmHg. GCS was 11/15 (E-3, V-3, M-5), and bilateral pupils were 3 mm in size and equally reacted to light. Fundoscopy did not reveal papilledema. The capillary blood glucose level was 130 mg/dl.

The patient was managed symptomatically with face mask O_2_ of 10 l/min. He was catheterized, and an input–output chart was generated. A 16G NGT was inserted, and he was treated with activated charcoal. Over 90 min, 3% saline 150 ml was given to alleviate possible cerebral edema. Blood was sent for investigations including venous blood gas. The latter yielded normal acid–base status, electrolytes, and lactate. An electrocardiogram revealed sinus tachycardia with a rate of about 120 bpm. X-ray of the chest was normal. The patient’s GCS gradually deteriorated, and simultaneously airway patency was reduced with stridor. He was intubated under direct laryngoscopy following rapid sequence induction with IV propofol 2 mg/kg and IV suxamethonium 2 m/kg and cricoid pressure by an anesthetist. The pharynx appeared inflamed. Intravenous dexamethasone 8 mg stat dose was administered. Arterial blood gas after the intubation revealed a pH of 7.43, PCO_2_ 27 mmHg, PO_2_ 241 mmHg with lactate of 4.3 mmol/l and base excess of −5 mmol/l. A 500 ml 0.9% saline bolus was administered, and the maintenance fluid was increased to 120 ml/h.

The patient was admitted to the intensive care unit (ICU) for further management and monitoring. His initial blood investigations are illustrated in Table 1.

**Table 1:** Initial blood investigations

Investigation	Results	Reference
Total white cell count	10.94 × 10^9^/µl	4–11
Neutrophils	59%	50–80
Lymphocytes	31%	30–40
Hemoglobin	13.1g/dl	11–14
Hematocrit	42.4%	35–45
Platelet count	322 × 10^9^/µl	15–450
C-reactive protein	4.4 mg/l	< 6
Creatinine	1.06 mg/dl	0.7–1.3
Serum sodium	131 mmol/l	135–145
Serum potassium	3.7 mmol/l	3.5–5.5
Serum urea	15 mg /dl	6–24
PT	10.2 s	15
INR	0.89	< 1.5
APTT	28.5 s	33
AST	179 U/l	8–33
ALT	196 U/l	7–56
Total bilirubin	0.9 µmol/l	1.8–20

PT: Prothrombin time, INR: International normalised ratio, APTT: Activated partial thromboplastin time, AST: Aspartate aminotransferase, ALT: Alanine Transaminase

**Fig. 1:**
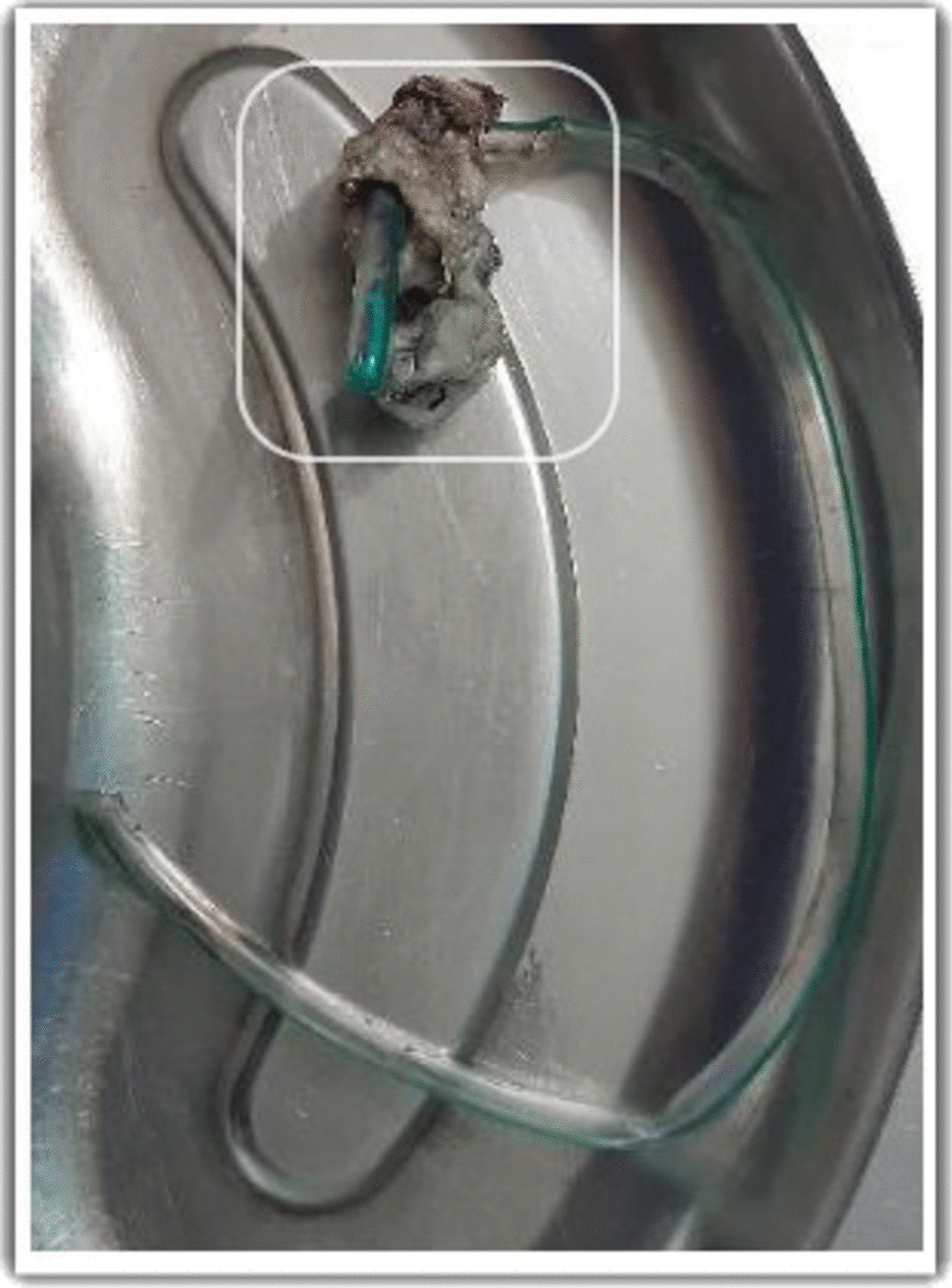
The clump of solvent cement (illustrated in white) formed at the distal end of the NGT (image taken after removal)

At the ICU, the patient was sedated and paralyzed to carry out lung and neuroprotective ventilation. Supportive care was given with stress ulcer and thromboprophylaxis. ENT referral was performed to assess the laryngeal and pharyngeal areas under fiber-optic laryngoscopy (FOL), and the findings were compatible with chemical laryngitis with mildly inflamed glottis, epiglottis, mildly edematous subglottic area, and vestibule of the larynx. Regular intravenous dexamethasone was administered. The patient was kept nil by mouth on the first 2 days, and NG feeding commenced after 48 h. His liver enzymes gradually dropped to normal levels. On day 3, sedation was stopped. The GCS had improved to 10/10. Successful extubation of the trachea was performed. Repeat FOL revealed that the laryngopharyngeal edema had now resolved. The patient appeared anxious and did not tolerate the NGT. As the patient was tolerating clear fluids via the oral route, the decision was made for its removal. Removal failed as the NGT was found to be lodged in the nasopharynx and attempts at pushing it back toward the oropharynx were unsuccessful. The patient became extremely anxious not responding to routine doses of sedatives. For the safety of the patient and the staff, he was reintubated and sedated. Subsequently, the NGT was removed by the ENT surgeon; the procedure was mildly traumatic with mild bleeding from the nasopharynx. The nasopharynx was packed with an adrenaline-soaked gauze. A Foley catheter was inserted, and the bulb was kept inflated for tamponade effect. The distal end of the NGT had formed a clump after reacting with *S*, which had led to the initial failure in removal (Fig. 1). The ENT team’s advice was to keep the pack for 48 h, after which the ENT team could remove the pack and the catheter. FOL was repeated, and inflammation of the laryngopharyngeal area had improved with the absence of significant bleeding. The leak test was repeated and found to be positive.

The nasal pack was removed after 48 h, and the patient’s trachea was extubated. Oral feeding was gradually commenced. He was discharged to the ward on day 5. A psychiatry referral was done at the ward, and upper gastrointestinal (GI) endoscopy was arranged at a tertiary care center in 2 weeks. Toxicology studies were not carried out as these were not available freely in this low-resource setting.

## Discussion

*S* is classified as a hazardous substance and Schedule 5 poison according to the manufacturer [1]. It consists of polyvinyl chloride polymer, and the main constituent is 1-cyclohexanone (25–70%) with a mixture of 3-butanone (1–15%) and 1-acetone (0–10%). Doses and plasma concentrations leading to toxicity have been studied in animals, while relevant human data for the product as a whole are not available [1]. It is found to cause toxic effects after exposure in humans that include dermal, eye, pulmonary, gastrointestinal, and nervous systems [2]. Cyclohexanone toxicity is rare in humans and animals used for experimental purposes [3]. Inhalation is the predominant route of toxicity and can lead to respiratory tract irritation and skin, eye irritation, and CNS depression in large quantities [4, 5]. Toxicity after ingestion of cyclohexanone-containing organic solvents has not been reported elsewhere, according to our knowledge, thus data on definitive management are lacking.

It is possible that our patient (initially managed in the emergency treatment unit) developed upper airway obstruction (indicated by the onset of stridor) owing to upper airway inflammation caused by *S* and subsequently developed hypoxia and hypercarbia resulting in reduced consciousness. The arterial blood gas analysis was performed after intubation, mechanical ventilation, and stabilization of the patient, which might not reflect the preceding parameters. Similarly, CNS depression directly due to the toxicity of *S* can also be a causative factor, although the risk is stated to be low following ingestion route [1].

According to the manufacturers, following acute ingestion, active vomiting is discouraged and measures should be taken to prevent aspiration in a patient who is vomiting [1]. Supportive therapy in the form of supplementary oxygen and correction of electrolytes is similarly advocated. Activated charcoal adsorbs nonpolar, poorly water-soluble compounds [6]. *S* is described as a partially miscible substance in water, thus the use of activated charcoal is justified in case of significant ingestion. As witnessed in our patient, any clinical features of respiratory tract inflammation and airway involvement indeed warrant heightened alertness for early identification and management of life-threatening compromise. In animal studies, cyclohexanone has been implicated in delayed liver, renal, and hematological derangements. It is thus prudent to monitor human exposures for an extended period.

*S* is commercially used as a bonding material for PVC tubing [1]. Interestingly, the NGT used in this patient was made of PVC. The ingested *S* likely led to the amalgamation of the distal end of the NGT. Owing to multiple distal side openings in the NGT, it would have been functional until removal. We came across a recent case report of a young male who presented with small bowel obstruction 8 months after ingestion of a chlorinated PVC solvent cement and was found to have an ileal perforation during open laparotomy [7].

## Conclusion

*S* poisoning following ingestion is seldom reported in literature. Clinicians need to be aware of the related potential airway and CNS emergencies. We present herein probably the first ever case of poisoning following ingestion of *S*. Initial resuscitation, continued in-hospital monitoring, and multidisciplinary care resulted in successful management of uncommon and unanticipated clinical encounters and importantly a favorable outcome.

## Data Availability

Data sharing does not apply to this article as no datasets were generated or analyzed during the current study.

## Abbreviations

*S*: S-lon®
PVC: Polyvinyl chloride
CNS: Central nervous system
NGT: Nasogastric tube
ENT: Ear, nose, throat
GCS: Glasgow Coma Score
FOL: Fiber-optic laryngoscopy
